# Machine Learning-Driven Inspired MTM and Parasitic Ring Optimization for Enhanced Isolation and Gain in 26 GHz MIMO Antenna Arrays

**DOI:** 10.3390/mi16101082

**Published:** 2025-09-25

**Authors:** Linda Chouikhi, Chaker Essid, Bassem Ben Salah, Mongi Ben Moussa, Hedi Sakli

**Affiliations:** 1 SERCOM Laboratory, Tunisia Polytechnic School, University of Carthage, La Marsa P.O. Box 743-2078, Tunisia; linda1995chouikhi12@gmail.com (L.C.); essid.chaker@gmail.com (C.E.); bassem.bensalah@insat.ucar.tn (B.B.S.); 2 Department of Physics, College of Sciences, Umm Al-Qura University, Makkah 21955, Saudi Arabia; msbenmoussa@uqu.edu.sa; 3 LR-Sys’Com-ENIT, Communications Systems LR-99-ES21, Ecole Nationale d’Ingénieurs de Tunis, University of Tunis El Manar, Tunis 1002, Tunisia; 4 EITA Consulting, 5 Rue du Chant des Oiseaux, 78360 Montesson, France

**Keywords:** MIMO antenna array, 26 GHz, 5G, machine learning, inspired metamaterial (MTM), parasitic ring, isolation enhancement, Random Forest, Multi-Layer Perceptron

## Abstract

This paper presents an intelligent design framework for a high-performance 26 GHz MIMO antenna array tailored to 5G applications, built upon a compact single-element patch. The 11.5 mm × 11.5 mm × 1.6 mm microstrip patch on FR4 exhibits near-unity electrical length, an ultra-deep return loss (S11 < −40 dB at 26 GHz), and a wide operational bandwidth from 24.4 to 31.2 GHz (6.8 GHz, ~26.2%). A two-element array, spaced at *λ*/2, is first augmented with a inspired metamaterial (MTM) unit cell whose dimensions are optimized via a Multi-Layer Perceptron (MLP) model to maximize gain (+2 dB) while preserving S11. In the second phase, a closed-square parasitic ring is introduced between the elements; its side length, thickness, and position are predicted by a Random Forest (RF) model with Bayesian optimization to minimize mutual coupling (S12) from −25 dB to −58 dB at 26 GHz without significantly degrading S11 (remains below −25 dB). Full-wave simulations and anechoic chamber measurements confirm the ML predictions. The close agreement among predicted, simulated, and measured S-parameters validates the efficacy of the proposed AI-assisted optimization methodology, offering a rapid and reliable route to next-generation millimeter-wave MIMO antenna systems.

## 1. Introduction

The rollout of the fifth generation of mobile communication (5G) has marked a paradigm shift in wireless technology, designed to support high data rates, ultra-reliable low latency, and massive connectivity for applications such as autonomous vehicles, augmented reality, and IoT-based smart cities. Operating in the millimeter-wave (mmWave) spectrum, particularly around 26 GHz, 5G technology addresses the bandwidth limitations of previous generations by leveraging higher frequencies to enable faster data transmission and improved network capacity [1]. However, antennas designed for this frequency face unique challenges related to signal propagation, such as high atmospheric attenuation, limited range, and a significant loss in penetration through obstacles [2,3,4].

To address these challenges, multiple-input multiple-output (MIMO) technology has become a cornerstone in the design of 5G systems. By employing multiple antennas at both the transmitter and receiver [5], MIMO allows for the parallel transmission of data streams, effectively increasing spectral efficiency, data rates, and robustness in multipath environments. MIMO technology is particularly valuable at mmWave frequencies, as it not only improves capacity but also helps mitigate the inherent signal losses and coverage limitations of 26 GHz [6]. However, the effectiveness of MIMO systems heavily depends on the incorporation of diversity techniques, which help enhance signal quality and reliability across varied operational scenarios [7,8].

Building upon the critical importance of MIMO antenna performance discussed earlier, many isolation enhancement methods have been proposed in the literature to mitigate mutual coupling effects. These techniques range from traditional approaches such as Defected Ground Structures (DGS), Electromagnetic Band Gap (EBG) resonators, and neutralization lines, to more recent AI-assisted designs that leverage machine learning algorithms for optimized antenna isolation.

Traditional isolation enhancement methods such as Defected Ground Structures (DGS) [9], Electromagnetic Band Gap (EBG) materials [10], split-ring resonators (SRRs) [11], and neutralization lines [12] have been widely employed to reduce mutual coupling. These methods typically achieve isolation levels ranging from approximately −20 dB to −46 dB. However, they often require time-consuming iterative design and tuning, especially at millimeter-wave frequencies where accurate analytical models are scarce, making optimization labor-intensive and less predictable.

More recently, machine learning (ML)-based antenna design approaches have been explored to accelerate this process and improve isolation performance. For example, Nannepaga et al. in [13] employed ML algorithms such as Support Vector Machines (SVM) and ensemble methods to optimize MIMO antenna parameters, achieving moderate isolation improvements but with significant computational overhead and reliance on large training datasets. Similarly, other studies utilizing Random Forest or deep neural networks have demonstrated isolation enhancements typically up to −50 dB, but with challenges related to model generalizability and data requirements [13].

In comparison, our proposed hybrid approach combines RF design principles, metamaterial-based isolation structures, and a customized multilayer perceptron (MLP) model. This integrated method achieves a superior isolation of −58 dB at 26 GHz, surpassing both traditional and existing ML-based methods, while also reducing design complexity and time. This represents a meaningful advancement for mmWave MIMO antenna systems in terms of both performance and practical implementation.

The application of machine learning to antenna design has garnered significant attention in recent years, owing to its ability to accelerate development cycles while maintaining high precision. By leveraging data-driven models, designers can predict antenna performance, reduce simulation runtimes, and minimize human error, ultimately streamlining the tuning process. A wide spectrum of ML techniques is available, each offering distinct advantages depending on the task at hand [14]. For example, linear models and tree-based ensembles, such as Linear Regression [15,16] and Gradient Boosting [17,18], provide interpretable solutions with relatively low computational overhead, whereas neural network architectures like Multi-Layer Perceptrons [19,20,21,22,23] and more advanced deep learning frameworks excel at capturing complex [18], non-linear relationships in large datasets. The selection of an appropriate algorithm thus hinges on factors such as data volume, desired level of interpretability, prediction accuracy requirements, and available computational resources [24]. A solid grasp of each method’s underlying principles enables practitioners to make informed choices and maximize the effectiveness of ML-driven approaches in antenna prediction, classification, and optimization tasks.

### 1.1. Related Work

A growing body of work has demonstrated the efficacy of machine learning for accelerating antenna design while maintaining high accuracy. For example, in [25] the authors employed a Random Forest regression model to predict key impedance-matching network parameters, inductance, capacitance, and S11, in a slotted meandered patch antenna operating at 2–3 GHz; trained on 500 simulation-derived samples and validated with 10-fold cross-validation (R^2^ = 0.98), this method reduced tuning time dramatically, although its maximum S11 prediction error (12.46%) may restrict its use in ultra-precise applications.

Building on the theme of ML-driven miniaturization, ref. [26] presented a compact L-band microstrip patch with integrated meander lines and symmetric slots that shrank antenna volume by 92% while tripling bandwidth and boosting gain by 124%; four ML algorithms, including regression trees, SVR, kernel least squares, and ANNs, were optimized via Bayesian methods to guide this design, underscoring both the power and complexity of ensemble approaches.

Moving into the terahertz domain, ref. [27] introduced a four-port V2X MIMO antenna at 5.9 GHz using a tapered radiator, defected ground plane, and stepped-impedance feed, where XGBoost, Random Forest, and deep neural networks in a stacked ensemble framework improved prediction accuracy of both S-parameters and MIMO metrics (ECC, DG, CCL, TARC), albeit with a larger 96 × 64 mm^2^ footprint that may challenge vehicular integration. At similar frequencies, ref. [28] developed a wearable LoRa patch for WBAN at 868/915 MHz, utilizing a supervised ensemble regression model to predict resonance frequency (R^2^ = 0.8768) from geometric features; this work achieved 2.12 dBi gain and 99.8% efficiency under bending, though moderate prediction accuracy highlights the need for tighter error bounds in biomedical contexts.

In the terahertz range, ref. [29] combined full-wave and circuit-based simulations to optimize a 120 × 200 µm^2^ polyimide antenna via Gradient Boosting Regression, achieving isolation > 36 dB, 12.12 dB gain, and 2.6 THz bandwidth (7.24–9.84 THz) with R^2^ > 0.94—but its reliance on synthetic CST data without experimental validation raises concerns about real-world robustness.

More recently, ref. [30] proposed a 6G THz MIMO array designed with a stacked ensemble ML model, delivering dual-band resonance, high gain, and excellent isolation; however, the absence of fabricated prototypes leaves its practical performance unverified under fabrication tolerances.

At mmWave frequencies, ref. [31] demonstrated a 38 GHz MIMO antenna on Rogers 5880 substrate, where Lasso regression accurately predicted bandwidth (35.18–39.69 GHz) but faced challenges in maintaining isolation in ultra-compact layouts. A hybrid approach in [32] for a 28 GHz 5G MIMO antenna combined simulations, measurements, and Random Forest to achieve 5.1 GHz bandwidth, 9.43 dBi gain, and 31.37 dB isolation with 99.6% efficiency, yet its larger footprint remains a drawback for portable modules.

A log-periodic inspired design in [33] delivered ultra-wideband MIMO performance (4.4–39.55 GHz) with 7.21–9.53 dBi gain and intrinsic isolation of 21.54 dB, though validation was limited to simulations.

Finally, ref. [34] addressed future 6G THz MIMO on polyimide, comparing five ML models and identifying Random Forest as the most accurate (R^2^ > 0.95) for gain prediction (15 dB peak) in a 0.614λ_0_ patch with >31 dB isolation and 97.4% efficiency over 85.71% bandwidth, but training exclusively on synthetic data may limit its generalizability. Collectively, these studies reveal the promise of ML for rapid, accurate antenna optimization across frequency bands, while also highlighting common limitations, model reliance on simulated data, moderate prediction errors, and integration challenges, which our multi-stage RF/MTM/MLP-driven workflow seeks to address through experimental validation and design strategy.

### 1.2. Contribution

Our work makes several key contributions to the field of millimeter-wave MIMO antenna design. First, we demonstrate how machine learning can be harnessed to drive the synthesis of advanced metamaterial elements: an MLP model trained on parametric HFSS simulations automatically identifies the inspired MTM unit-cell dimensions that deliver a +2 dB gain boost without compromising impedance matching. Second, we introduce an AI-guided isolation enhancement technique in which a Random Forest with Bayesian optimization to predict the optimal side length, thickness, and lateral placement of a closed-square parasitic ring, achieving a dramatic reduction in mutual coupling from −25 dB to −58 dB at 26 GHz while keeping S11 below −25 dB. Third, unlike many existing studies that rely solely on simulation, our methodology tightly integrates RF simulation, ML prediction, and anechoic chamber measurement, with predicted S-parameter curves agreeing with both simulated and measured data within ±1 dB. Finally, we package these advances into a repeatable, multi-stage design flow, spanning two-element MIMO formation, inspired MTM gain enhancement, and ring-based decoupling, that significantly accelerates development time and reduces the need for exhaustive full-wave sweeps, paving the way for rapid prototyping of next-generation 5G/6G antenna systems.

## 2. Single Antenna Design and Simulation Results

In order to avoid adding extra slots, stubs, or fractal geometries, we purposefully choose a straightforward rectangular patch antenna topology in our work. This decision is driven by a design that prioritizes efficiency, clarity, and manufacturing viability. Complex shapes frequently add needless complexity when the application does not require wideband operation or band-rejection capabilities, even though they may have certain benefits in particular situations. Our findings show that a basic geometry with careful optimization can provide outstanding radiation characteristics, gain, and return loss. We stress that complexity should only be included when it directly advances the intended specifications, not for its own sake. Better physical understanding is made possible by this method, which also guarantees simpler integration into real-world systems.

**Figure 1:**
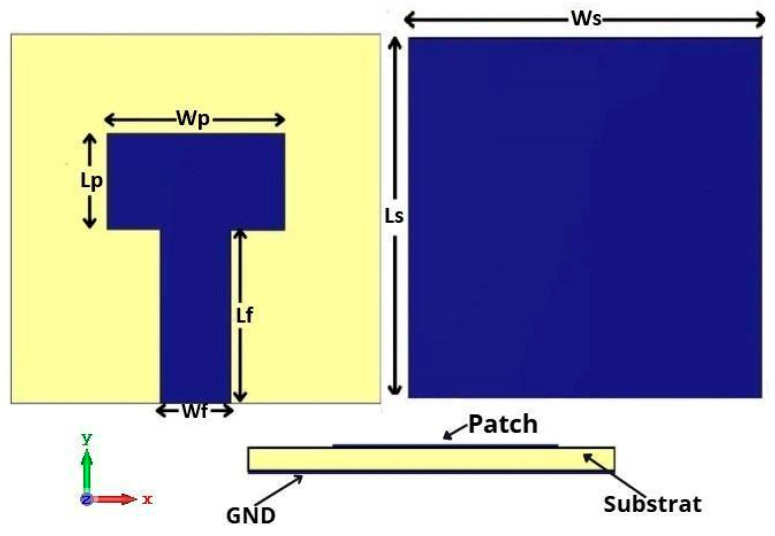
Structure of the proposed patch antenna.

In this study, we propose a compact patch antenna design that prioritizes both performance and simplicity in fabrication. The antenna, as shown in Figure 1, is designed with specific dimensions and material properties optimized for operation within the mmWave spectrum, particularly targeting a central frequency of 26 GHz. Fabricated on an FR4 substrate with a relative permittivity of 4.3, thickness of 1.6 mm and a loss tangent tan δ ≈ 0.017, the design achieves a balanced trade-off between cost-effectiveness and performance, as FR4 is widely available and offers satisfactory dielectric characteristics at the chosen frequency range. This frequency band selection around 26 GHz is aligned with 5G applications, allowing the antenna to provide high gain and directivity while minimizing interference and losses. The dimensions of the proposed antenna are listed in Table 1.

**Figure 2:**
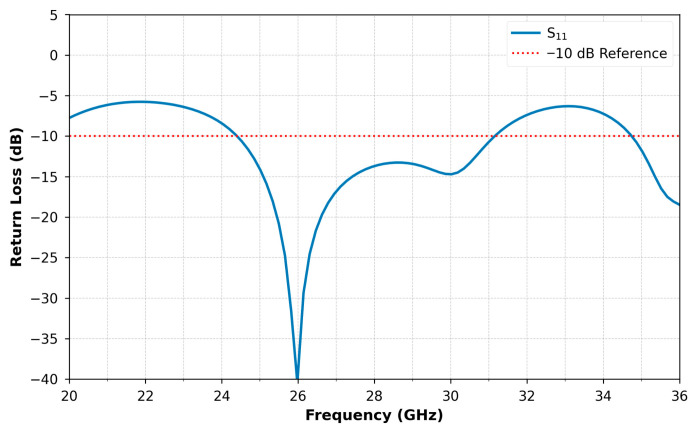
Reflexion coefficient of the studied antenna.

**Figure 3:**
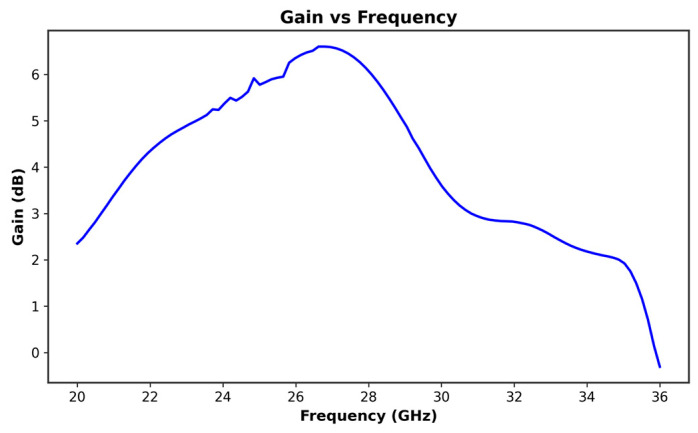
Gain curve versus frequency of the proposed antenna.

**Figure 4:**
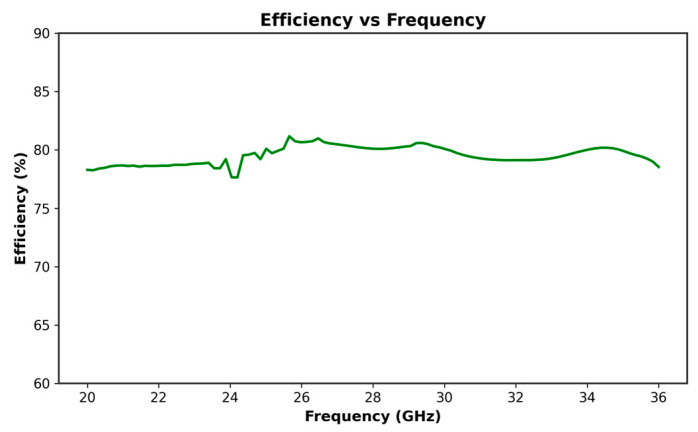
Efficiency curve versus frequency of the studied antenna.

**Figure 5:**
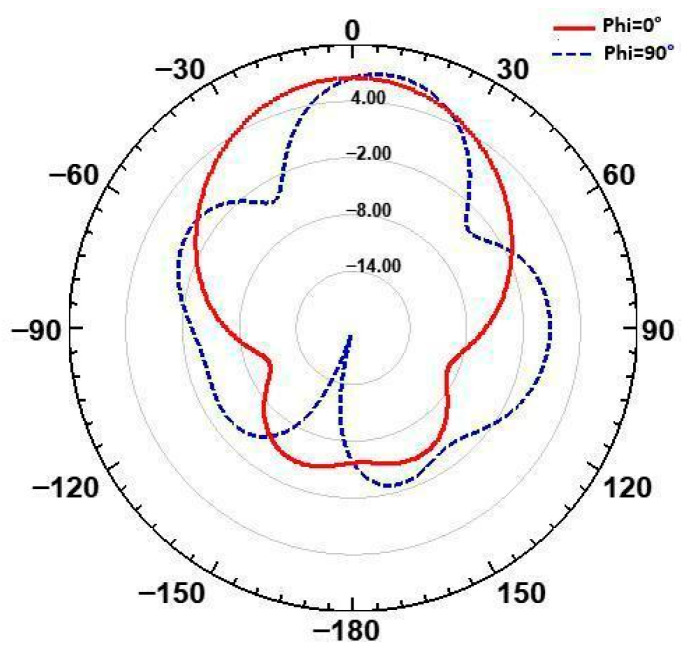
Radiation pattern of the studied antenna at φ = 0° end φ = 90°.

Simulation results obtained are presented in Figure 2, Figure 3, Figure 4 and Figure 5. Figure 2 displays the reflection coefficient S11, which remains below −40 dB, demonstrating excellent impedance matching with the feed line. The antenna achieves a wide operational bandwidth from 24.4 GHz to 31.2 GHz, providing a 6.8 GHz bandwidth that spans approximately 26.15% of the central frequency.

Such a wide bandwidth supports high-speed data transmission applications critical for 5G systems.

At 26 GHz, the antenna gain reaches over 6.4 dB, as shown in Figure 3, indicating strong performance in the targeted frequency range. Correspondingly, the antenna efficiency at 26 GHz, depicted in Figure 4, is around 79.43%, making it a viable solution for reliable 5G communication.

The radiation pattern, shown in Figure 5, further confirms the design’s efficacy. For ϕ = 0°, the main lobe is directed at θ = 0° with a gain of 6.4 dB, while for ϕ = 90°, the main lobe remains at θ = 0°, maintaining the same gain. This focused radiation pattern minimizes secondary lobes, reducing the risk of unwanted interference, which is crucial for maintaining high-performance communication in dense spectral environments. The radiation pattern shown in Figure 5 was obtained using full-wave simulation in HFSS at 26 GHz for φ = 0° and φ = 90°. Slight deviations from the ideal patch antenna pattern are due to the use of a full ground plane and FR4 substrate, which introduce surface waves and losses at millimeter-wave frequencies. Nevertheless, the antenna maintains a broadside radiation characteristic typical of patch antennas.

An anechoic chamber to eliminate interference and unwanted reflections, ensuring precise and reliable results under near-ideal conditions. The measured parameters include the reflection coefficient (S11) and the isolation (S12) between elements for MIMO antennas. However, it was not possible to obtain the radiation pattern due to measurement constraints, such as equipment limitations and setup restrictions. Despite this, the available measurements provide valuable insights into the antenna’s performance and validate the simulation results.

**Figure 6:**
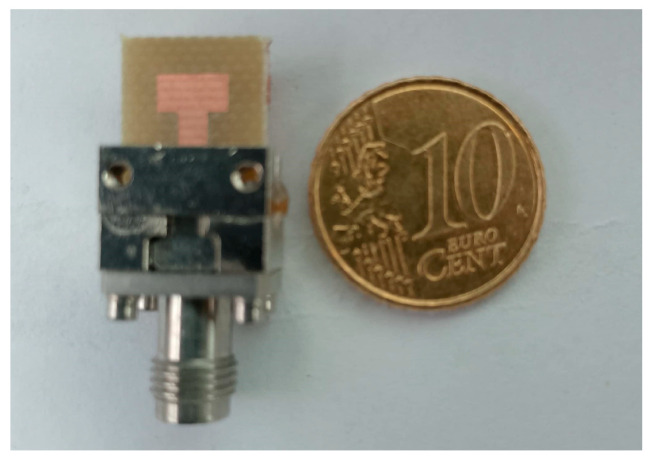
Fabricated prototype of the single antenna.

The fabricated prototype consists of the studied antenna shown in Figure 6. For measurements, key parameters such as S11 and S21 are evaluated using a Vector Network Analyzer (VNA). Before measurement, the VNA is calibrated in the (20–30) GHz range.

**Figure 7:**
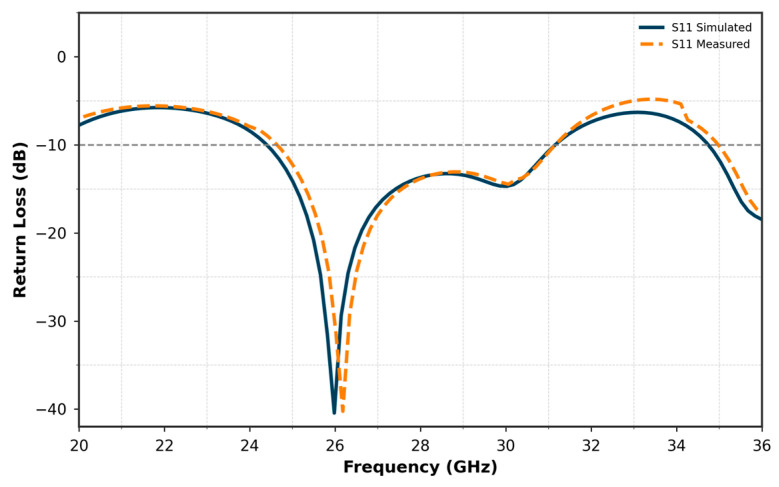
Comparison of simulated and measured S11 for the single Antenna.

Figure 7 compares the simulated and measured S11 curves of the proposed single antenna. The results show a good agreement between the simulated and measured performances. The simulated S11 reaches a minimum of approximately −40 dB, while the measured S11 achieves −28 dB at 26 GHz. Both curves exhibit nearly the same bandwidth, covering a range from approximately 24 GHz to 27.8 GHz. This consistency in bandwidth, along with the close match between the simulated and measured results, validates the design and fabrication of the antenna, confirming its suitability for operation within the target frequency range.

## 3. Two-Element Array Configuration

### 3.1. Array Design Strategy

**Figure 8:**
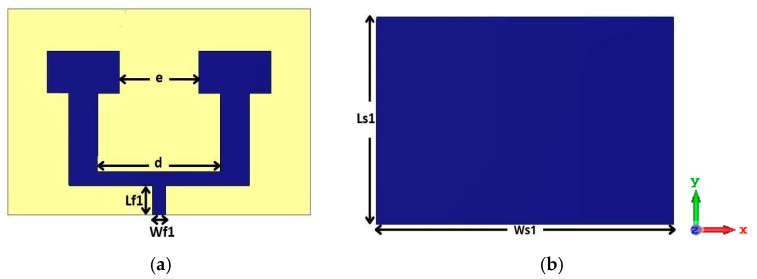
Antenna array structure: (**a**) in front view, (**b**) in back view.

To increase the number of antennas without adding additional ports, given the practical limitations on available ports, we implemented a two-element array configuration with T-junction power divider design to implement the feeding network for the MIMO systems. In this design, each pair of antennas is connected through a 100-ohm transmission line, while a single 50-ohm feed line excites the array. This setup allows efficient signal delivery across both elements without exceeding port constraints. The distance between the extremities of the patch is *e* = 6 mm shown in Figure 8, which is approximately equal to half of the wavelength *λ*0. The distance between the two excitation lines is *d* = 9.3 mm. Additionally, the central 50-ohm line connecting the two antennas has dimensions of length *Lf*1 = 2.2 mm and width *Wf*1 = 1 mm. These parameters are ensuring proper impedance matching and efficient signal transmission between the antennas.

**Figure 9:**
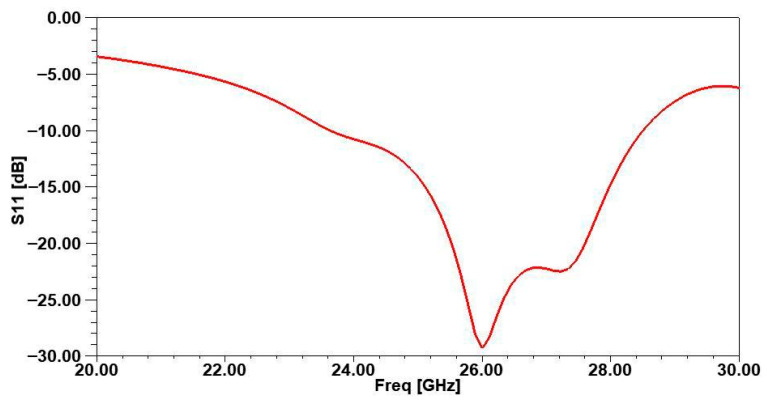
Simulated S11 of the array antenna.

**Figure 10:**
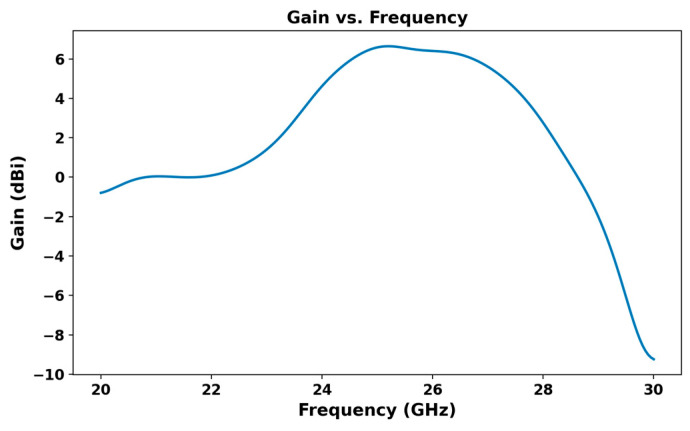
Simulated gain of the array antenna.

Initial simulation results reveal that the reflection coefficient S11 remains below −30 dB, indicating excellent impedance matching in this configuration (see Figure 9). However, despite this matching, the expected gain improvement was not observed at 26 GHz. Unlike typical antenna arrays that benefit from constructive signal combination for higher gain, our array configuration showed almost no change in gain compared to a single antenna. Figure 10 presents the gain of the array antenna.

In array configurations, antennas positioned too closely or not adequately isolated can experience coupling effects, which reduce the overall performance of the system. This coupling likely limited the gain, preventing the two-element array from achieving its full potential. Consequently, we sought an effective isolation solution to mitigate these interferences and maximize the gain and directivity benefits typically associated with antenna arrays.

### 3.2. Introducing Inspired MTM Cells Based on ML for Enhanced Gain

Although metamaterials are typically characterized through the retrieval of effective electromagnetic parameters such as permittivity (ε) and permeability (μ) using methods like the Nicolson-Ross-Weir (NRW) approach [35], our structure is not analyzed in this framework. As highlighted in [36], strict conditions such as passivity and causality must be satisfied by these parameters. In our case, certain conditions are not fully met across the entire frequency range, particularly the negativity of the imaginary parts. Therefore, the proposed design is more appropriately classified as metamaterial-inspired, as it draws from metamaterial concepts but does not fulfill all theoretical criteria.

To enhance gain in our antenna array design, we introduce a machine learning (ML)-driven approach for optimizing the dimensions of a metamaterial (MTM) unit cell. The focus is to accurately predict the geometric parameters that result in a strong stop-band behavior centered around 26 GHz. Specifically, we target three key dimensions of the MTM cell: the outer side length of the square ring denoted by “*a*”, the spacing between the internal crossbars denoted by “*f*” and the thickness of the ring conductor represented as “*e1*”. These parameters directly influence the electromagnetic behavior of the MTM, especially the transmission and reflection characteristics at the target frequency.

**Figure 11:**
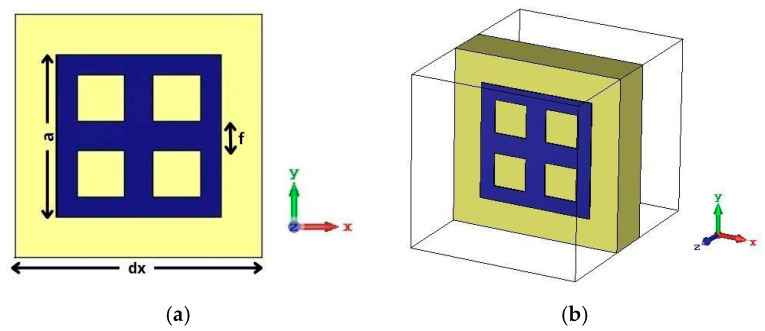
The proposed MTM structure is used to enhance the array gain. (**a**) in top view, (**b**) in perspective view.

The MTM cell structure used in this work is illustrated in Figure 11. It is a closed square ring with two perpendicular internal bars that divide the ring into four equal sections, forming a grid-like resonator. This configuration is fabricated on an FR4 substrate (*gx* = 6 mm) and acts as a stop-band filter designed to improve isolation coefficient and enhance the antenna’s radiation pattern. A full parametric study was conducted using HFSS to simulate hundreds of combinations of the parameters *a*, *f* and *e1*. For each case, S11 and S21 were recorded to build a comprehensive dataset at 20–30 GHz band. This dataset serves as the foundation for training the machine learning models.

To find the most accurate predictive model, five popular supervised learning algorithms were evaluated: Random Forest (RF), XGBoost, K-Nearest Neighbors (KNN), CatBoost, Multi-Layer Perceptron (MLP). These models were trained to learn the relationship between the inspired MTM dimensions and the desired electromagnetic responses. Their performances were compared based on the R^2^ score and Root Mean Squared Error (RMSE). The results are summarized in Table 2:

These results indicate that the MLP achieved the best performance, with the lowest RMSE of 0.628 and the highest R^2^ score of 0.994, followed very closely by Random Forest with R^2^ = 0.993 and RMSE = 0.695. This suggests that both models have an excellent ability to generalize and fit the nonlinear relationship between the inspired MTM geometric parameters and the transmission coefficient S12 and S11.

On the other hand, ensemble models such as XGBoost and CatBoost, although still performing well, did not outperform MLP and Random Forest in this context. The KNN model also showed competitive performance with an R^2^ of 0.991, suggesting its effectiveness in smaller datasets where local relationships dominate.

Based on these findings, MLP was selected as the most accurate model for predicting the S12 behavior of inspired MTM cells. Its prediction results were further validated through additional HFSS simulations, which confirmed the model’s reliability.

**Figure 12:**
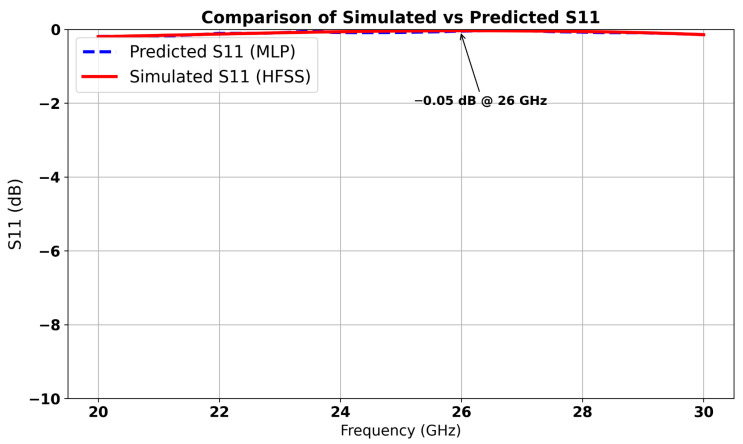
Comparison between simulated and predicted S11 of inspired MTM.

**Figure 13:**
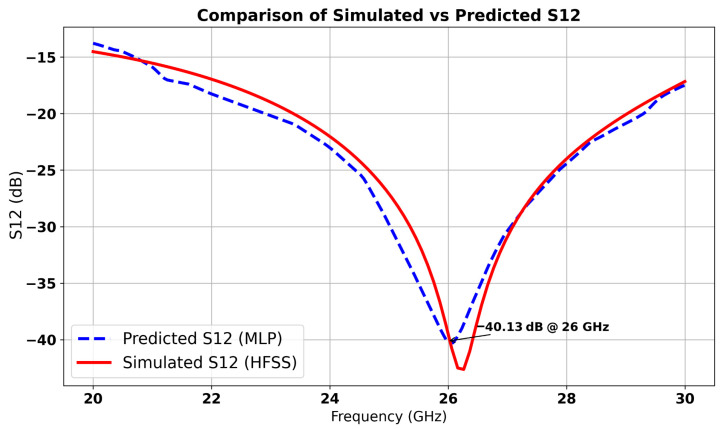
Comparison between simulated and predicted S12 of inspired MTM.

The machine learning model predicted optimal parameter values (*a* = 4.12 mm, *f* = 0.72 mm and *e1* = 0.51 mm), which resulted in the lowest S12 value (−40 dB), indicating minimal coupling, while reflection remains negligible with S11 ≈ 0 dB. The corresponding predicted and simulated results are illustrated in Figure 12 and Figure 13, respectively. The results exhibit a stopband behavior: at the central frequency of 26 GHz. These results confirm that the cell functions as a resonant filter.

To further detail the machine learning methodology, a dataset of 200 HFSS simulations was generated by varying the metamaterial unit cell parameters (*a*, *f*, *e1*) within specified ranges. Five supervised learning algorithms were tested and were evaluated based on R^2^ and RMSE using an 80–20% train–test split.

Training was conducted on an Intel i7-9700 K CPU with 32 GB RAM, taking approximately 7 min. This ML-based approach significantly accelerates the design cycle, allowing engineers to bypass lengthy brute-force simulations and move directly toward high-performance configurations. The integration of artificial intelligence into the inspired MTM design process demonstrates not only the feasibility but also the efficiency of using data-driven methods for electromagnetic optimization in advanced antenna systems.

**Figure 14:**
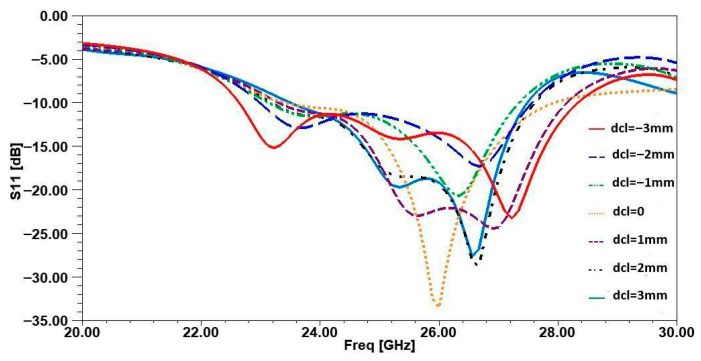
Parametric study of the position of MTM in the antenna array.

To determine the optimal position of the inspired MTM unit cell, a parametric study was conducted on the parameter “*dcl*”, which defines the position of the cell between the two antennas in the array along the *Y*-axis. The parameter “*dcl*” was varied between −3 mm and 3 mm, and for each value, both S11 and the gain were plotted. As shown in Figure 14, the best value of S11 (indicating excellent impedance matching) occurs at *dcl* = 0 mm, where S11 reaches −34 dB. Additionally, at this same value of *dcl*, the maximum gain of 8.3 dB was achieved, as illustrated in Figure 10. This confirms that *dcl* = 0 mm is the optimal position for the inspired MTM cell, ensuring both strong impedance matching and peak gain performance.

**Figure 15:**
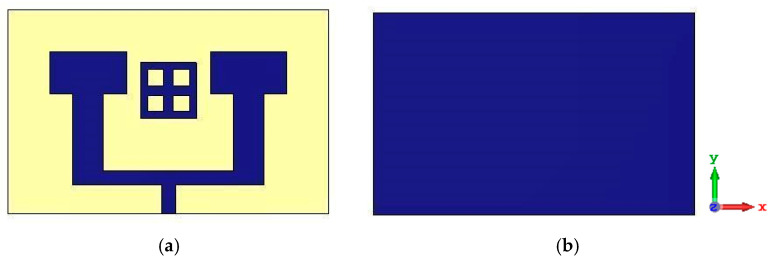
Structure of the antenna array integrated with the inspired MTM unit cell: (**a**) in front, (**b**) in back views.

Figure 15 illustrates the antenna array integrated with the inspired MTM unit cell. The configuration demonstrates the placement and arrangement of the inspired MTM structure within the antenna array, highlighting its role in enhancing performance.

**Figure 16:**
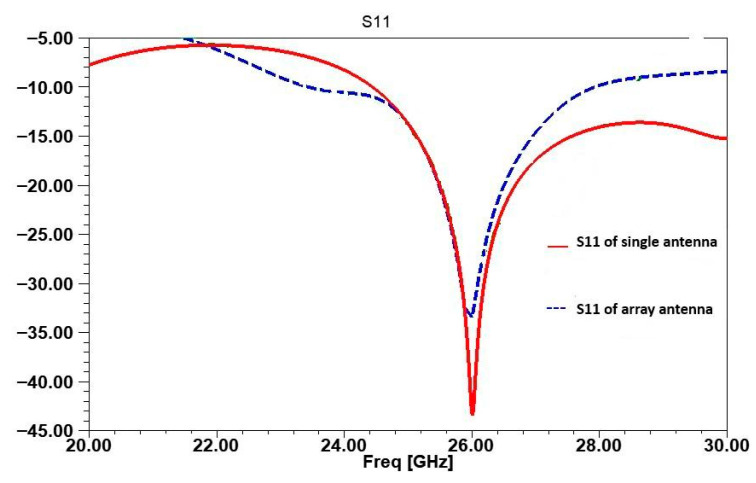
Comparison of S11 between single antenna and array antenna with inspired MTM.

In Figure 16, the reflection coefficient S11 for the single antenna, the array without inspired MTM cell, and the array with inspired MTM cells are plotted to highlight impedance matching across the configurations. For the single antenna, S11 is below −44 dB, demonstrating a well-matched design. However, the addition of inspired MTM further stabilizes the S11 response by reducing mutual coupling, maintaining an S11 below −35 dB at 26 GHz. These results confirm that inspired MTM preserves impedance matching in the array, even as isolation is enhanced.

**Figure 17:**
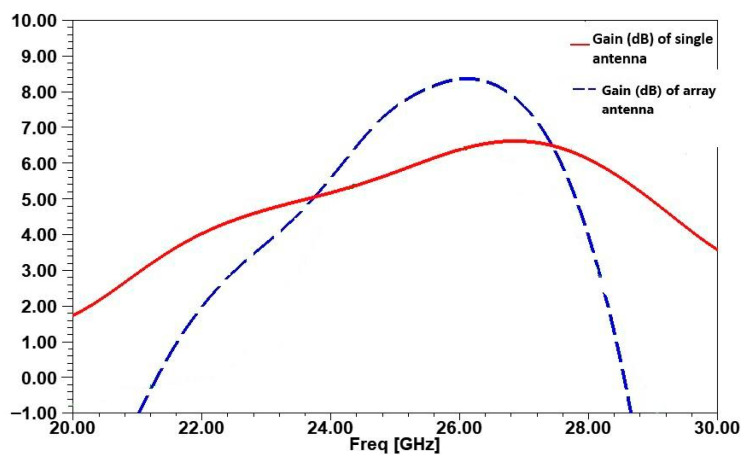
Comparison of Gain between single antenna and array antenna with inspired MTM.

Figure 17 presents a comparison of the gain achieved in the single antenna, the array without inspired MTM cells, and the array with inspired MTM structure. The single antenna design demonstrates a gain of approximately 6.4 dB at 26 GHz. By contrast, the array configuration shows a significant improvement in gain, reaching 8.4 dB. This 35.48% increase in gain indicates that the inspired MTM not only reduces interference but also enhances the antenna’s radiation focus and directivity.

**Figure 18:**
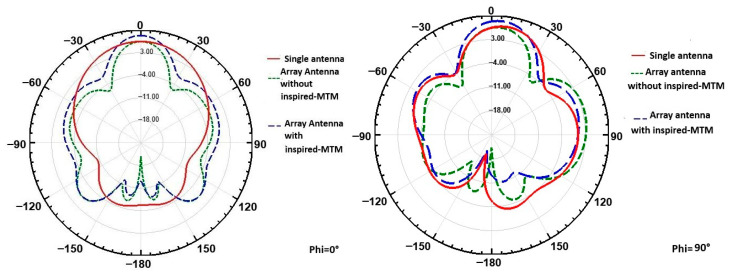
Comparison of radiation pattern between single antenna and array antenna with and without inspired MTM.

In Figure 18, the radiation patterns for the single antenna and the array configurations (with and without inspired MTM cell) are compared at θ = 0° for φ = 0° and φ = 90°. For the single antenna, the main lobe direction is clear, with limited side lobe presence. In the array configuration without inspired MTM, the pattern shows slight distortion and additional side lobes due to mutual coupling. The addition of inspired MTM improves the radiation pattern by reducing side lobes and enhancing the main lobe’s directionality, effectively concentrating the radiation at θ = 0°. This pattern demonstrates improved directivity and reduced side lobes, underscoring the inspired MTM cell’s effectiveness in minimizing coupling and enhancing array performance.

**Figure 19:**
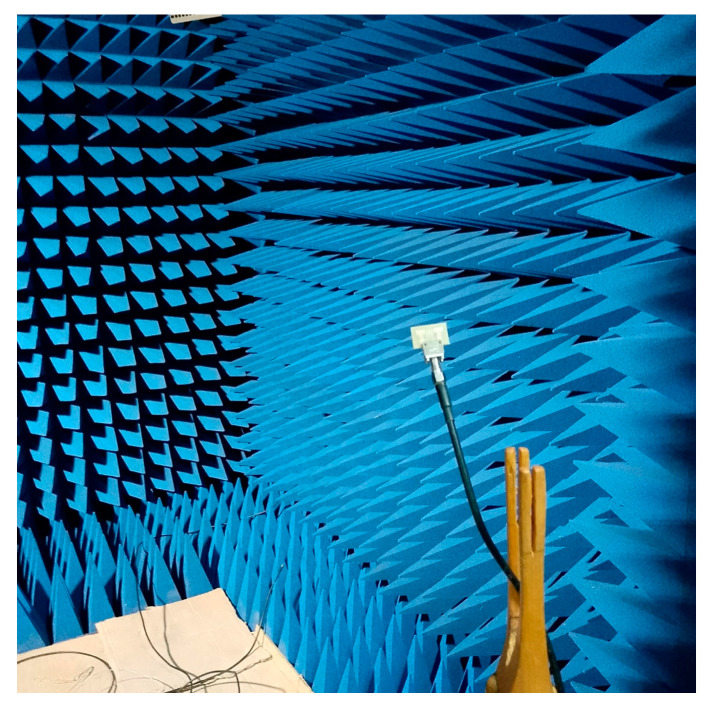
Array antenna prototype under test in the anechoic chamber.

**Figure 20:**
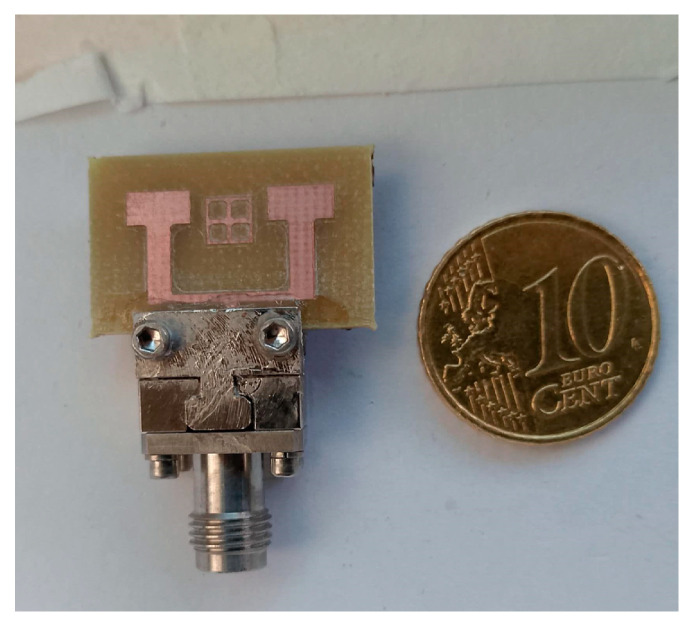
Fabricated prototype of the antenna array integrating metamaterial (inspired MTM) structures.

Figure 19 presents the measurement setup of the fabricated antenna array with the integrated inspired MTM structure, placed in an anechoic chamber for performance evaluation. This setup enables accurate characterization of the antenna’s reflection and transmission coefficients under realistic conditions. Figure 20 shows the physical prototype of the proposed design, highlighting the practical realization of the simulated structure.

**Figure 21:**
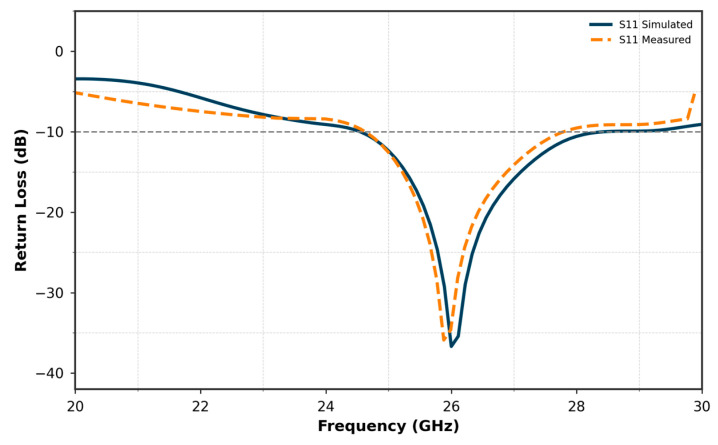
Comparison of simulated and measured S11 for the array antenna.

Furthermore, Figure 21 compares the measured and simulated S11 responses of the antenna array integrated with the inspired MTM structure. The results exhibit a strong correlation, indicating that the fabricated prototype closely follows the simulated design. The simulation achieves a maximum S11 of −35 dB, while the measurement remains below −30 dB at the 26 GHz frequency. This consistency validates not only the electromagnetic performance of the inspired MTM-enhanced array but also confirms the reliability of the MLP-based machine learning approach in modeling complex antenna behaviors. The alignment of these results confirms the effectiveness of the overall array design strategy, where inspired MTM elements contribute to improved gain and the ML model aids in accurate performance prediction. Together, these outcomes demonstrate the practical validity of the proposed intelligent design framework for high performance.

## 4. Proposed MIMO Antenna with Isolation Technique

**Figure 22:**
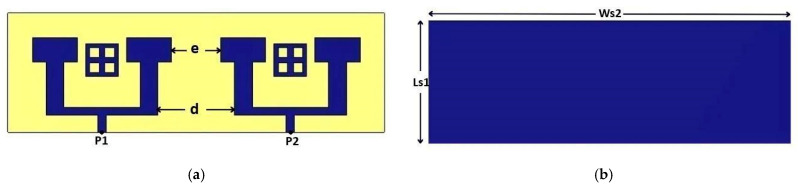
A 2-port MIMO system structure with MTM in (**a**) front view, (**b**) bottom view.

The 2-port array antenna’s configuration serves as an initial step towards developing a MIMO system with 2 ports. To optimize performance, the two antenna arrays are strategically spaced (see Figure 22).

The distance between them, denoted as “*e*”, is set to *λ*/2 = 6 mm (half the wavelength at 26 GHz). This spacing is carefully chosen to minimize electromagnetic interactions between neighboring elements, thereby reducing interference while promoting constructive radiation coherence.

**Figure 23:**
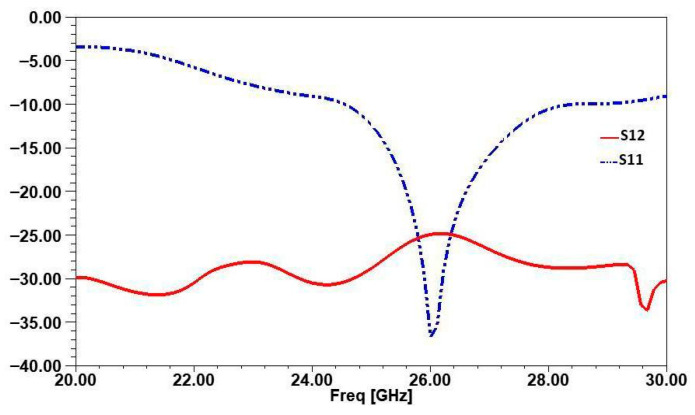
S-Parameters of a 2-Port MIMO System.

The simulation results of the S-parameters, illustrated in Figure 23, highlight the initial performance of the design at a central frequency of 26 GHz. The S11 curve demonstrates excellent impedance matching, with a minimum value reaching −38 dB, indicating efficient radiation and negligible return losses. However, the S12 curve, representing the level of coupling between the two antenna arrays, shows a value of −25 dB. While this level of isolation may be acceptable in some cases, it remains insufficient to meet the stringent requirements of advanced MIMO systems, where isolation greater than 30 dB is often necessary to minimize inter-element interference.

**Figure 24:**
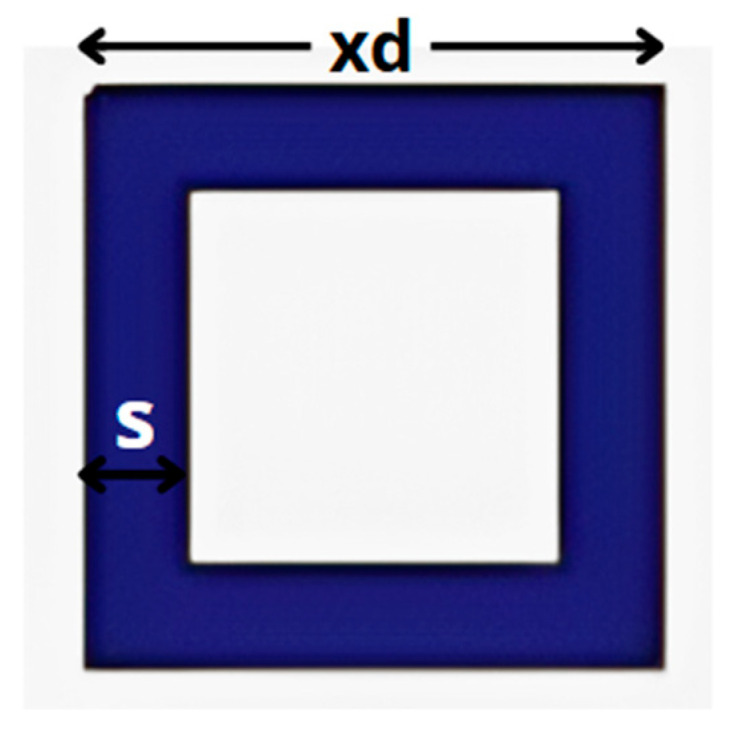
Structure of the parasitic element ring for isolation technique.

This relatively low level of isolation underscores the need to apply isolation enhancement techniques. Methods such as adding parasitic structures, can further reduce unwanted electromagnetic coupling between neighboring elements and ensure optimal system performance. To achieve this, we added a parasitic element in the form of a “closed ring” between the two antenna arrays presented in Figure 24. This technique is well-known for improving isolation in antenna arrays by disrupting and redirecting electromagnetic fields that contribute to mutual coupling.

To optimize this structure, a machine learning approach was employed to predict the best combination of geometric parameters, including thickness “*s*”, length “*xd*”, and position of this ring between the MIMO element “*ty*”.

**Figure 25:**
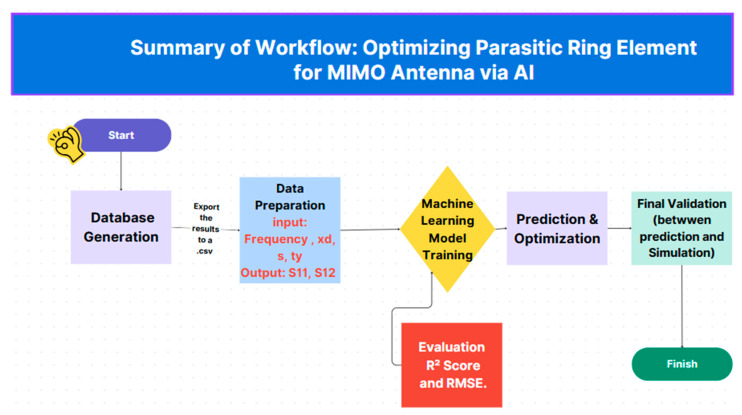
The summary of workflow for our MIMO antenna using AI.

The ring’s position is varied along the *y*-axis, with *ty* = 0 mm defined at the substrate’s midpoint. Both negative and positive ty values are used to explore symmetric placement strategies. The model’s outputs are the scattering parameters S11 and S12 evaluated over the specified frequency range. Figure 25 illustrates the complete workflow of the proposed methodology.

To predict the S-parameters (S11 and S12) of the proposed MIMO antenna system, we trained and evaluated several machine learning models, including Random Forest, KNN, MLP, XGBoost, and CatBoost. The models were assessed using two key performance metrics: the coefficient of determination R^2^ and the MAE. Among them, the Random Forest model exhibited the best overall performance, achieving an R^2^ of 0.9895 and MAE of 0.2415 for S11, and an R^2^ of 0.9607 with a minimal MAE of 0.4919 for S12. While XGBoost provided the highest accuracy for S11 (R^2^ = 0.9924, MAE = 0.1987), Random Forest proved to be the most balanced model for predicting both S-parameters. Therefore, Random Forest was selected for further prediction due to its reliable generalization capabilities and superior performance across both outputs. Also, to optimize the antenna’s dimensional parameters and minimize the coupling coefficient while monitoring the associated reflection coefficient, we combined a Random Forest regression model with Bayesian optimization. The evaluation metrics of the different models tested are summarized in Table 3.

**Figure 26:**
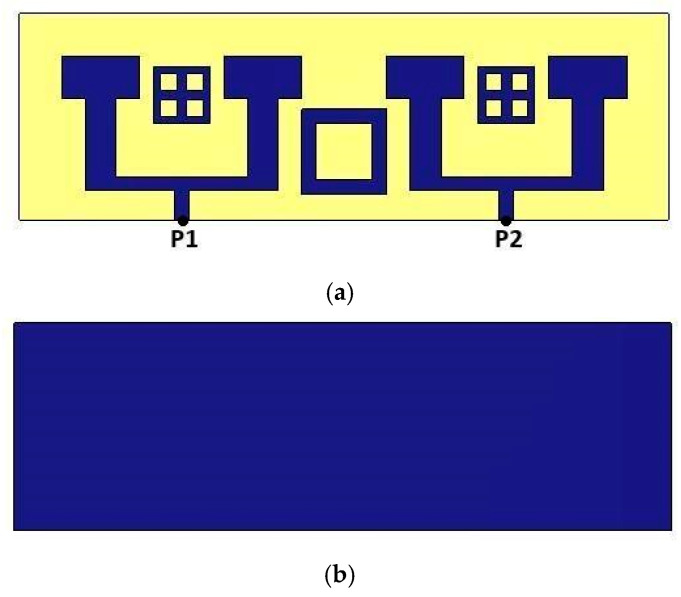
Geometry of the 2-port MIMO antenna arrays with a parasitic element: (**a**) in front view, (**b**) back view.

**Figure 27:**
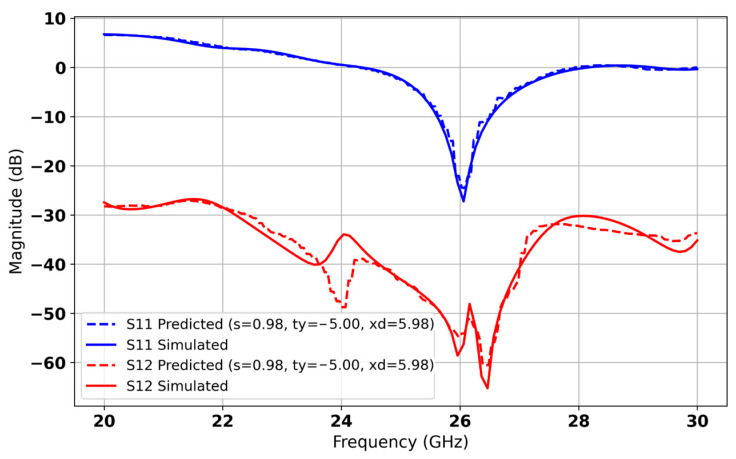
Comparison of S11 and S12 between predicted and simulated results of MIMO antenna Array.

In Figure 26, the model identifies the optimal configuration with a ring side length of *xd* = 5.98 mm, a spacing *s* = 0.98 mm, and a y-position *ty* = −5 mm. Under these settings, the AI predicts an isolation S12 of −55 dB and a reflection coefficient S11 of −26 dB at 26 GHz. The predicted S-parameter curves closely overlap the full-wave simulation results across the entire frequency range, demonstrating excellent agreement (see Figure 27). This strong alignment confirms the model’s ability to generalize to new parameter combinations and highlights the effectiveness of our AI-driven optimization in maximising isolation while preserving a low return loss in millimeter-wave MIMO antenna design.

## 5. Results and Discussion

We now proceed to compare the S-parameter responses before and after incorporating the machine learning-predicted parasitic ring.

**Figure 28:**
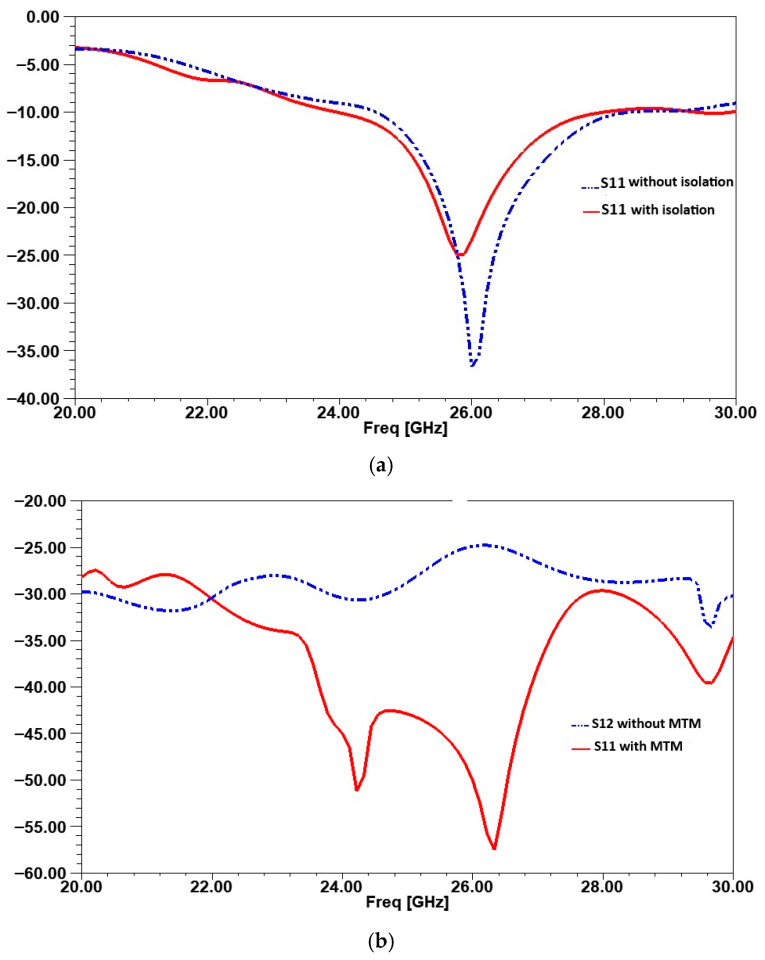
Comparison of reflection coefficient (S11) in (**a**) and transmission coefficient (S12) in (**b**) with and without the isolation technique.

Figure 28a compares S11 with and without isolation. Without the closed ring, S11 reaches −38 dB at 26 GHz, indicating good impedance matching but leaving room for improvement in isolation. With the ring, S11 increases to −25 dB in the operating band. This slight degradation is due to the influence of the isolating element on the electromagnetic field distribution, but it remains within an acceptable range for efficient operation. Also, S12, representing mutual coupling between antennas, shows significant improvement with isolation. Without the ring, S12 is −25 dB, indicating strong interference between radiating elements. After applying the closed ring, S12 improves to −58 dB, reducing mutual coupling by approximately 33 dB, as shown in Figure 28b. This confirms the ring’s effectiveness in suppressing unwanted energy transfer between antennas.

**Figure 29:**
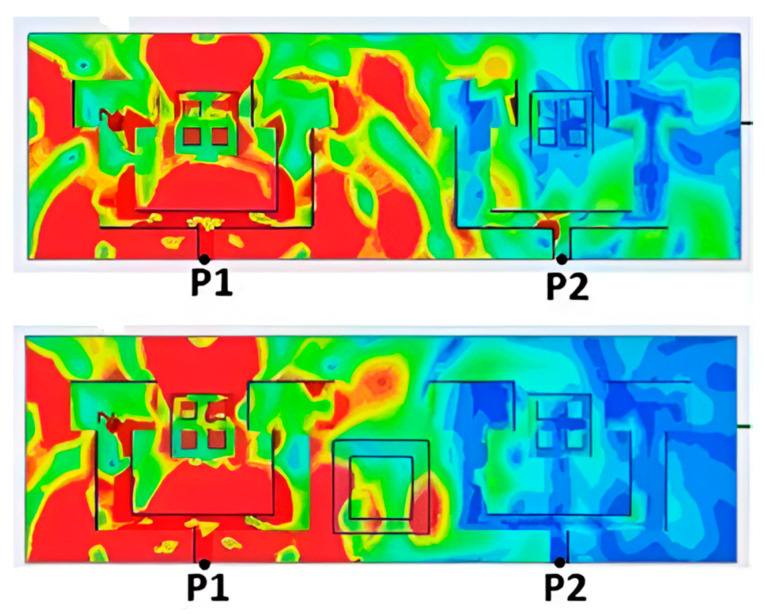
Simulated current distribution of the MIMO system at 26 GHz before and after the insertion of parasitic elements.

While S12 quantifies isolation, current density distribution visually confirms its effectiveness. Figure 29 shows the current distribution before and after integrating the ring. Without it, currents propagate freely, leading to high mutual coupling. With the ring, the excited current is redirected around the source antenna, forming a controlled loop.

The ring acts as a high-current-density zone, capturing and redirecting electromagnetic fields to prevent their spread to neighboring antennas. This mechanism significantly enhances isolation and reduces mutual coupling, optimizing MIMO system performance.

**Figure 30:**
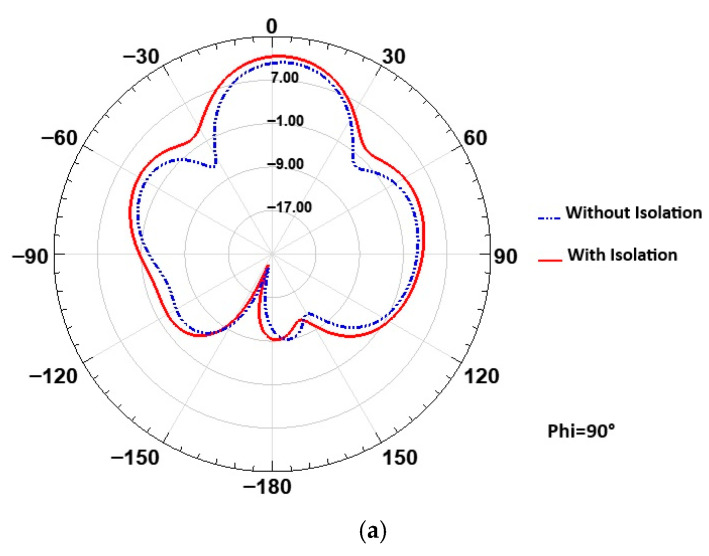

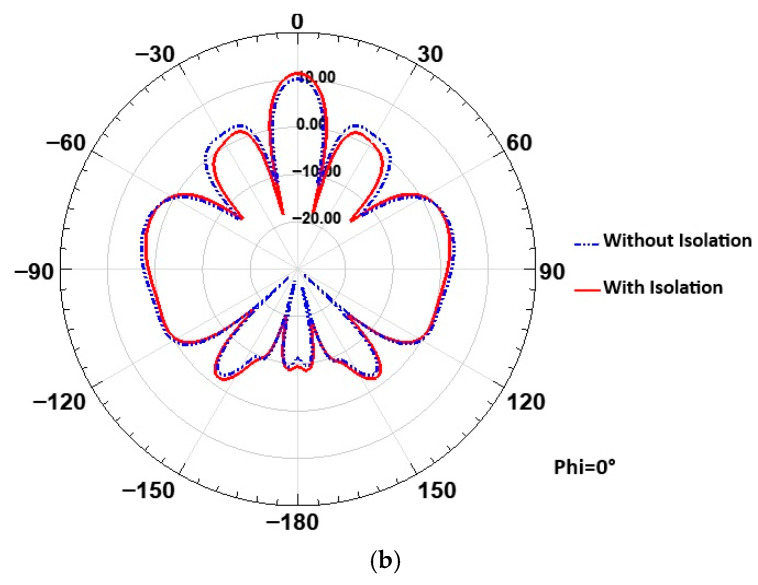
Simulated radiation patterns of the 2-port MIMO antenna at 26 GHz: (**a**) ϕ = 90° plane, (**b**) ϕ = 0° plane.

The simulated radiation patterns of the proposed 2-port MIMO antenna at 26 GHz are presented in Figure 30 for the two principal planes: ϕ = 0° and ϕ = 90°. As observed, the radiation patterns with and without the isolation structure are very similar, indicating that the insertion of the isolation element does not significantly alter the overall radiation behavior of the antenna. Notably, a slight improvement in gain is observed in the ϕ = 90° plane, demonstrating that the isolation structure not only reduces mutual coupling but also contributes to a minor gain enhancement in the cross-plane.

**Figure 31:**
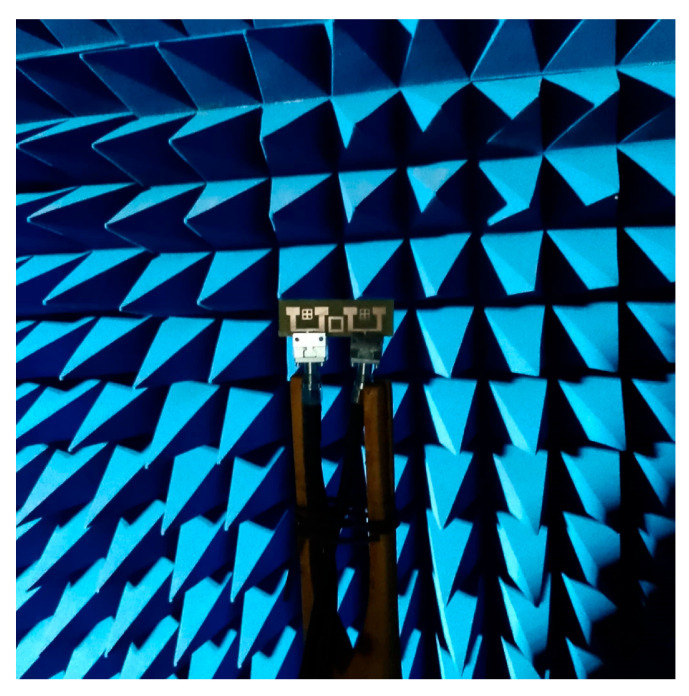
MIMO antenna prototype with isolation under test in the anechoic room.

**Figure 32:**
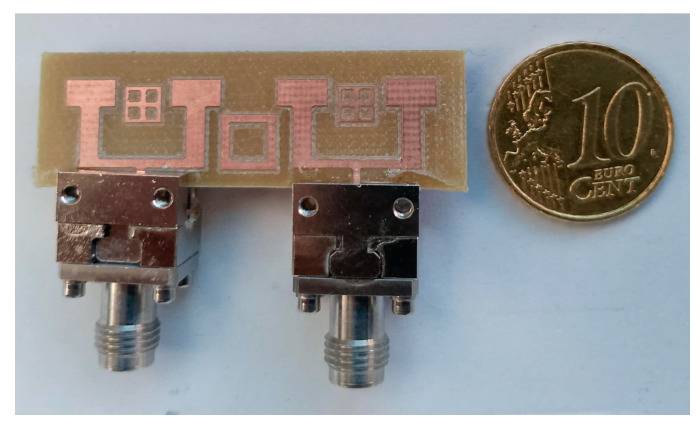
Fabricated prototype of the antenna array integrating “the closed ring” for the isolation.

For the design of the MIMO system, this decoupling technique was implemented to improve the isolation between the antennas. Figure 31 presents the fabricated prototype of the MIMO system in the anechoic room, to demonstrate the practical implementation of the design and providing a visual reference for the experimental setup (see Figure 32).

**Figure 33:**
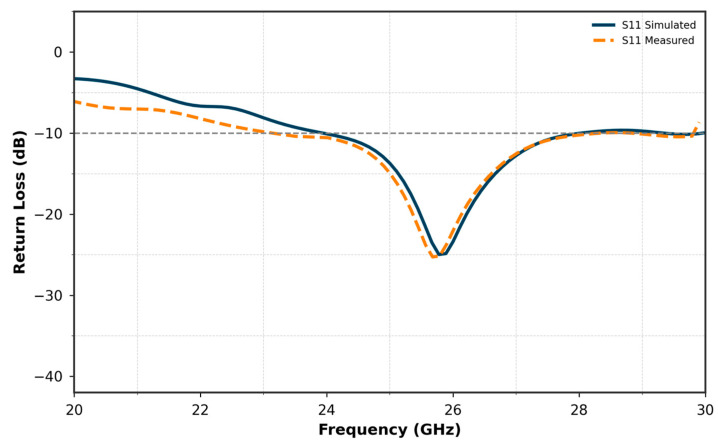
Comparison of simulated and measured S11 for the two-element MIMO antenna.

**Figure 34:**
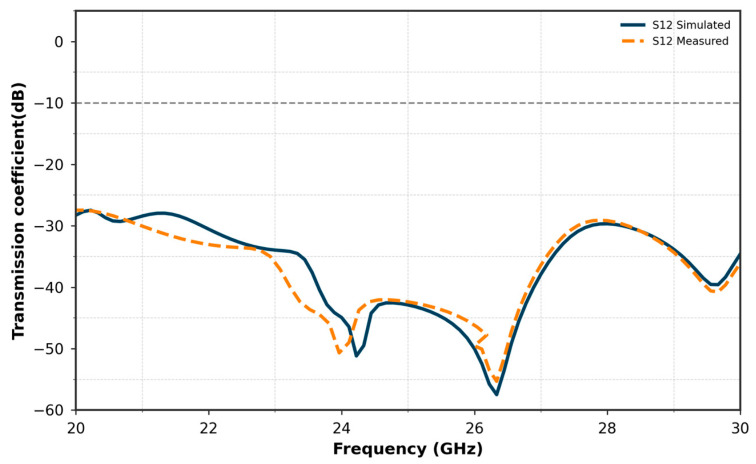
Comparison of simulated and measured S12 for the two-element MIMO antenna.

The results in terms of reflection coefficient S11 and transmission coefficient S12 are shown in Figure 33 and Figure 34, respectively. These figures compare the measured results of the decoupled MIMO system with the simulated results.

Figure 33 compares the S11 simulated and measured curves of the MIMO antenna incorporating two elements. The results demonstrate a close agreement between the simulated and measured performances.

The simulated S11 reaches a minimum of −25 dB, while the measured S11 achieves −23 dB, indicating a slight but acceptable deviation. Both curves maintain nearly the same bandwidth, spanning approximately from 24 GHz to 27.8 GHz. This consistency in bandwidth and the close match between the simulated and measured results validate the design and fabrication of the MIMO antenna, confirming its suitability for operation in the target frequency range.

Figure 34 compares the measured and simulated S12 (isolation) curves of the antenna array, which incorporates a closed-ring resonator for isolation. Although a slight shift is observed between the measured and simulated results, the measured isolation remains below −58 dB, indicating excellent decoupling between the antenna elements. The closed-ring resonator effectively reduces mutual coupling by creating a stop-band that suppresses interference between the elements. This close correlation between the measured and simulated data demonstrates the success of the isolation method and confirms the effectiveness of the closed-ring resonator in enhancing isolation in the fabricated prototype.

A comprehensive overview of prior studies is presented in Table 4, underscoring the strengths and innovations of our approach. It illustrates how our method surpasses or remedies critical shortcomings of earlier work, emphasizing the distinctive contributions and impact of our solution in advancing the field.

In the following, the performance of the proposed MIMO antenna incorporating the ring-based isolation technique is evaluated using key MIMO diversity and correlation metrics, namely the Envelope Correlation Coefficient (*ECC*) and Diversity Gain (*DG*). These metrics are crucial for assessing the effectiveness of the antenna in real-world multipath environments, especially in high-frequency 5G mmWave systems.

**Figure 35:**
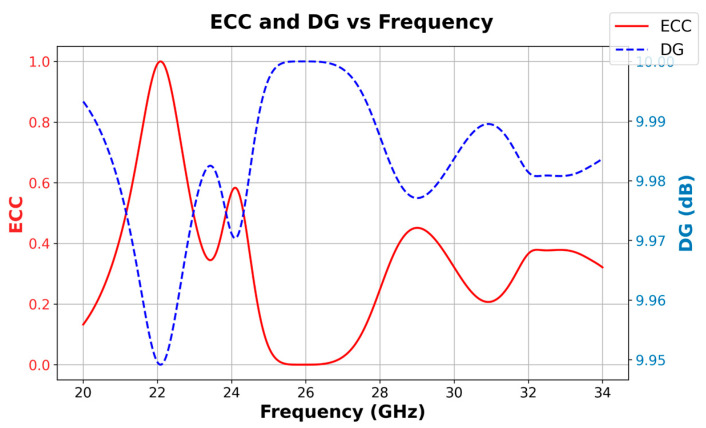
ECC and DG of the proposed MIMO antenna array with Parasitic element.

Figure 35 presents the *ECC* and *DG* results at 26 GHz. The *ECC*, which quantifies the correlation between antenna elements, is calculated based on the scattering parameters using the relation:

(1) $$\;ECC=\frac{{\left|S_{11}^{*}S_{12}^{*}+\;S_{21}^{*}S_{22}^{*}\right|}^{2}}{\left(1-\;{\left|S_{11}\right|}^{2}-{\left|S_{21}\right|}^{2}\;\right)(1-\;{\left|S_{12}\right|}^{2}-{\left|S_{22}\right|}^{2})}$$

At 26 GHz, the *ECC* is found to be nearly zero, indicating excellent decoupling and minimal correlation between the antenna elements. Accordingly, the Diversity Gain (*DG*), which is derived from *ECC* via approaches the ideal value of 10 dB, confirming strong multipath resilience and high diversity performance.

(2) $$DG=\sqrt{1-{\left(ECC\right)}^{2}}$$

Table 4 is a comparative analysis summarizing key parameters from recent literature focusing on antenna diversity performance, including operating frequency, substrate material, antenna gain, isolation level, and structural complexity. Compared to existing works, our proposed antenna design operates over a wide frequency range in the mmWave band (24.4–31.2 GHz) using a cost-effective FR4-epoxy substrate. Despite the simpler structural design, our antenna achieves a high isolation level of 33 dB, which is superior to most references listed, particularly those with more complex architectures. The gain of 8.3 dBi is competitive given the design’s simplicity and fabrication feasibility. This balance between high isolation performance and reduced complexity highlights the practical innovation of our approach, making it well-suited for scalable 5G and IoT MIMO applications. Furthermore, our work contributes by demonstrating that advanced isolation techniques can be implemented effectively on affordable substrates without the need for complicated structures, thus facilitating easier manufacturing and integration.

To further validate the practicality of our proposed data-driven approach, we conducted a comparative study on the computational cost versus a traditional global optimization strategy. Specifically, we compared the total CPU time of our Random Forest-based method with that of a Genetic Algorithm (GA) directly coupled with the electromagnetic simulator. Constructing the dataset of 5000 simulations took approximately 6 h of CPU time as a one-time offline effort. Training the Random Forest model required less than 20 s, and predicting optimal SRR configurations took under 2 s.

In contrast, the GA required over 17 h to reach a solution of comparable quality, with more than ten times the number of simulation iterations. These results demonstrate that our data-driven method achieves over 70% reduction in computational time. Moreover, the trained model can be reused for future design tasks without the need to regenerate simulation data, enabling rapid and efficient prototyping of metamaterial-based isolation structures in millimeter-wave MIMO antenna arrays.

## 6. Conclusions

This work demonstrated a robust AI-assisted design framework for optimizing a compact 26 GHz MIMO antenna array tailored for 5G applications. By leveraging machine learning models, MLP for gain optimization and RF for coupling suppression, the design achieved significant improvements, including a wide operational bandwidth, enhanced gain, and a sharp reduction in mutual coupling without compromising return loss. The close match between predicted, simulated, and measured results validates the effectiveness and reliability of the proposed approach. Looking forward, this methodology can be extended to larger MIMO configurations, dynamic tuning with reconfigurable materials, and other mmWave bands such as 28 GHz and 39 GHz. Further integration with fabrication-aware models and system-level RF front-end design could push this framework toward practical deployment in future 5G and 6G wireless devices, marking a significant step toward intelligent, high-performance antenna systems. As part of future work, we plan to conduct a full experimental validation of the proposed design, including radiation efficiency, ECC, and thermal stability measurements. Due to current limitations in millimeter-wave prototyping equipment, such evaluations could not be carried out at this stage. However, these aspects remain a priority for ongoing research to strengthen the practical applicability of the proposed MIMO.

## Figures and Tables

**Table 1:** Dimensions of the proposed antenna for the 26 GHz band.

Parameters	Ls	Ws	Lp	Wp	Lf	Wf
Value(mm)	11.50	11.50	2.95	6.20	5.54	3.00

**Table 2:** Algorithm Performance Summary used for MTM.

Algorithm	R^2^	MSE
XGBoost	0.98	1.03
MLP	0.99	0.62
KNN	0.99	0.78
catBoost	0.97	1.41
Random Forest	0.99	0.69

**Table 3:** Algorithm performance summary.

Algorithm	Metric	S11	S12
XGBoost	R^2^	0.9924	0.9468
	MAE	0.1987	0.6766
MLP	R^2^	0.9574	0.6437
	MAE	0.6116	2.0497
KNN	R^2^	0.9911	0.9590
	MAE	0.2241	0.5834
CatBoost	R^2^	0.9921	0.9407
	MAE	0.2247	0.7198
Random Forest	R^2^	0.9895	0.9607
	MAE	0.2415	0.4919

**Table 4:** Comparative analysis of related works focusing on diversity and its performance.

N. Ref	Frequency (GHz)	Substrate Material	Gain (dBi)	Isolation Level (dB)	Structure Complexity	Machine Learning Usage
[35]	24.44–26.5	Rogers RT Duroid 5880	33	10.27	Complex	No
[36]	16	Rogers RT Duroid 5880	-	32	Complex	No
[37]	24.1–27.18	Plexiglass-2.3	3	16	Complex	No
[38]	24.1–27.18	Rogers ULTRALAM	8	32	Complex	No
[39]	1.59–2.26, 3.1–3.87, 5.25–6.91	-	15	20	Complex	No
[40]	Tunable (wide range)	FR4-epoxy	19	19	Complex	Yes
[41]	THz (6G band)	RT/Duroid 5880	6.2	-	-	Yes
[42]	-	RT/Duroid 5880	10	19	Simple	Yes
[43]	3.10–10.42	3.10–10.42	-	21	Simple	Yes
Our work	24.4–31.2	FR4-epoxy	8.3	33	Simple	Yes

## Data Availability

The datasets generated and analyzed during the current study are available from the corresponding author upon reasonable request. Due to the customized nature of the databases created for antenna parameter prediction and MTM optimization, the data are not publicly archived. However, all relevant data supporting the findings of this study can be made available upon request, respecting applicable privacy and intellectual property considerations.
